# Accuracy and reliability of knee goniometry methods

**DOI:** 10.1186/s40634-018-0161-5

**Published:** 2018-10-19

**Authors:** Graeme Ethan Hancock, Tracey Hepworth, Kevin Wembridge

**Affiliations:** 1 Sheffield Teaching Hospitals NHS Foundation Trust, Sheffield, UK; 2 Rotherham General Hospital, Rotherham, UK

## Abstract

**Background:**

Measuring knee range of motion is important in examination and as a post-operative outcome. It is therefore important that measurements are accurate. Knee angles can be measured by traditional goniometers, smartphone apps are readily available and there are also purpose made digital devices. Establishing the minimum difference between methods is essential to monitor change. The purpose of this study was to assess reliability and minimum significant difference of visual estimation, short and long arm goniometers, a smartphone application and a digital inclinometer.

**Methods:**

Knee angles were assessed by 3 users: one consultant orthopaedic surgeon, one orthopaedic surgical trainee and an experienced physiotherapist. All 5 methods were used to assess 3 knee angles, plus full active flexion and extension, on 6 knees. The subjects had knee angles fixed using limb supports during measurement, whilst maintaining appropriate clearance to allow a reproduction of assessment in clinic. Users were then blinded to their results and the test was repeated. A total of 300 measurements were taken.

**Results:**

Inter-rater and intra-rater reliabilities were high for all methods (all > 0.99 and > 0.98 respectively). The digital inclinometer was the most accurate method of assessment (6° minimum significant difference). The long arm goniometer had a minimum significant different of 10°, smartphone app 12° and both visual estimation and short arm goniometry were found to be equally inaccurate (14° minimum significant difference).

**Conclusion:**

The digital inclinometer was the most accurate method of knee angle measurement, followed by the long arm goniometer. Visual estimation and short goniometers should not be used if an accurate assessment is required.

## Background

One of the key outcomes and measurable variables for any procedure around the knee is range of movement. 67° of flexion is required for a normal gait, 83° for ascending and 90° for descending stairs, 93° to stand from a seated position and 105° to tie shoes (Dietz et al., 2017). Full extension of the knee is also key to decrease quadriceps contraction and energy use for standing and walking. Range of motion is often measured after total knee arthroplasty, and indeed flexion of 90° is a requirement for discharge post-operatively in our unit and guides the need for further intervention is some instances. Accuracy of these measurements are therefore key, both for monitoring patient progress and for research.

Surgeons will often estimate range of motion visually in clinic. This is a quick and relatively easy method, but not typically accurate. Goniometers in both short and long-arm form are commonplace in the orthopaedic surgeon and physiotherapist’s armament for measuring joint angles, though logically a short-arm goniometer would not seem to give accurate results given the long lever around the knee that is being measured (and also the fact that the femur is a deep structure with a normal anterior bow). The advent of smartphones has led to the availability of numerous goniometer ‘apps’ using accelerometer technology to estimate angles, and many of these have been studied in the literature, with variable results (Cleffken et al., 2007; Lenssen et al., 2007; Pereira et al., 2017; Ferriero et al., 2013; Jones et al., 2014).

Previous studies do not describe a clinically useful statistical assessment for accuracy of goniometers, using only reliability tools, and studies assessing outcome based upon range of motion, have stated the use of ‘a goniometer’ only (Brosseau et al., 2001), and thus conclusions cannot be strongly made.

A useful method for joint angle measurement should have good inter and intra-rater reliability, and low potential error in measurement (minimum significant difference), but it is also key to be user-friendly and quick for use in the outpatient clinic.

The use of radiographs as a ‘gold standard’ (Edwards et al., 2004) for measurement is neither clinically effective, as it is resource heavy and results in unnecessary radiation exposure, and cannot necessarily be used reliably for measuring change in range of motion, and we therefore aim to determine the minimum significant difference between clinically useable devices to determine which device is most beneficial for clinical use and research.

We aim to identify inter and intra-rater reliability (validity of measurement) and minimum significant difference (accuracy/precision of measurement), thereby identifying the most accurate device for repeated clinical use for: visual estimation, short-arm goniometer, long-arm (50 cm) goniometer, smartphone app and a digital goniometer device called Halo Digital Goniometer.

Visual estimation is the estimate of knee angle without any measuring devices, a short-arm goniometer is a goniometer of 30 cm length in total with a central articulation with measurements in degrees, a long-arm goniometer with 50 cm arms and central articulation and measurement, a smartphone app which measured an angle between two screen presses and the Halo Digital Goniometer.

We hypothesise that the visual estimation and short arm goniometer techniques will be less reliable than the long arm goniometer and digital devices. We aim to establish the minimum difference required between measurements to be sure of a valid difference in measurement when using any of these devices.

## Methods

Ethical approval was granted by the Health Research Authority for this study. 3 healthy subjects, whom all had a normal body mass index, and no previous or current knee pathology or symptoms, consented to have their knee angles measured. Both knees were measured in each subject, non-sequentially, to reduce estimation bias. Each knee was measured twice, with a time interval of approximately 2 h between measurements of the same angle, to give data for an intra-rater reliability measurement. Anatomical landmarks were not marked in order to give reproducibility in the outpatient clinic.

**Fig. 1:**
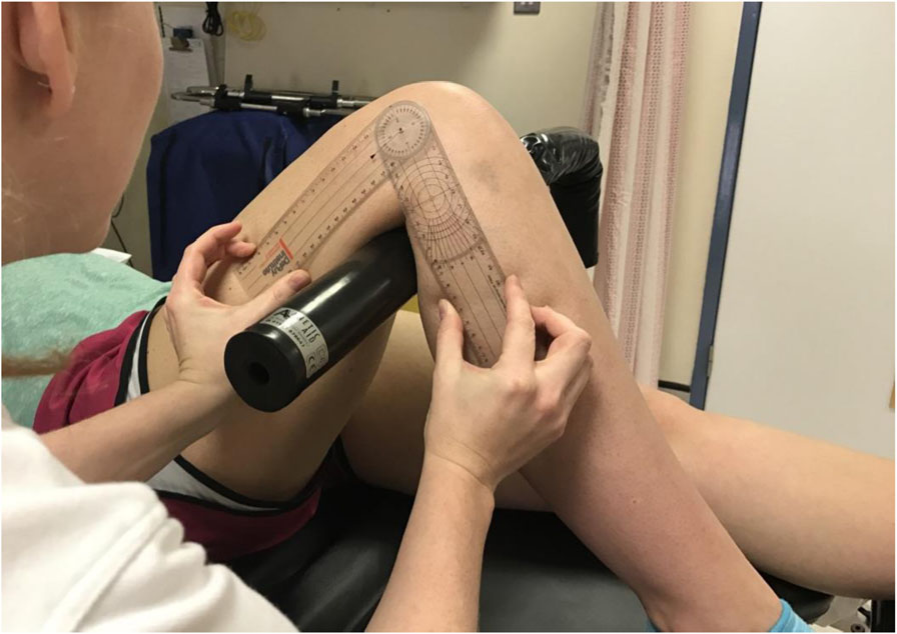
Use of a short arm goniometer for knee angle measurement (subject supine on an operating table with a 90 degree support under the knee)

Measurements were taken with each subject supine on an operating table. A limb support with a 90° bend was attached to the table, and placed in the popliteal fossa to support the knee and maintain an angle for all measurements to be taken (Fig. 1). The height of this was placed at random, for 3 separate heights. The ankle position was not fixed, in order to prevent subject discomfort and allow all measurements to be taken as they would be in clinic, but the position of the lateral malleolus relative to the table was marked and checked regularly during measurement to ensure no significant change in position.

**Fig. 2:**
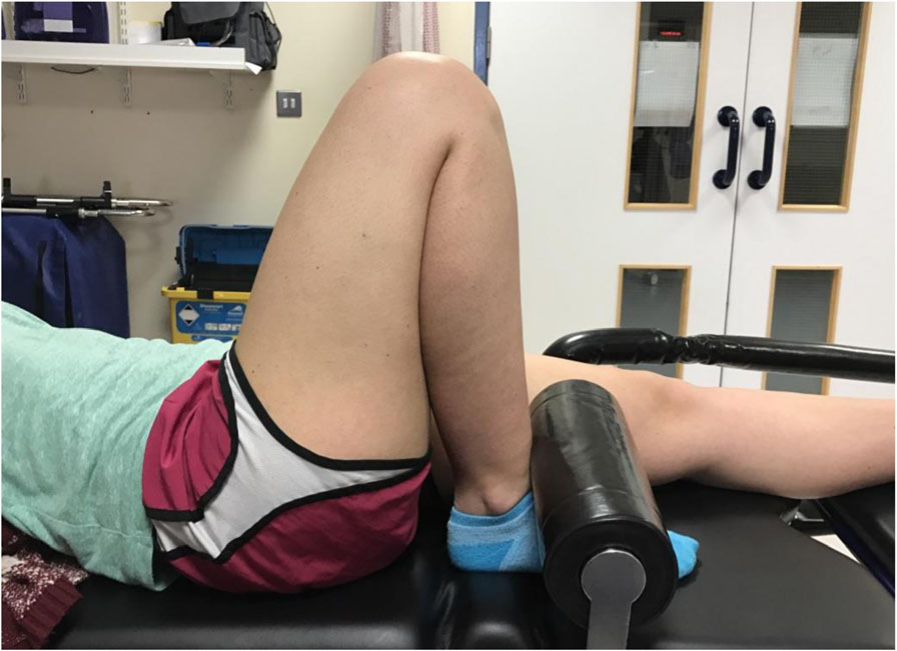
Technique for measuring maximal flexion

**Fig. 3:**
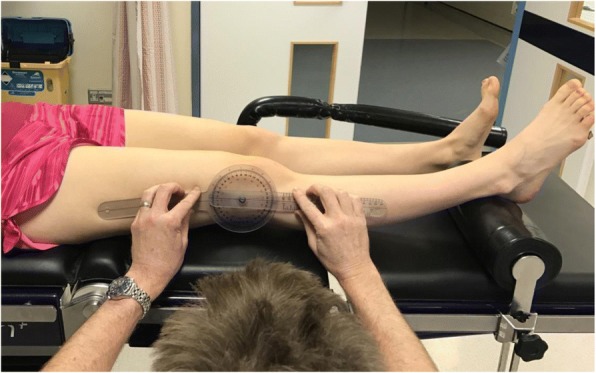
Technique for measuring full extension (long arm goniometer used)

At each position (for the first measurement of each knee) the distance between the centre of the greater trochanter and the centre of the lateral malleolus was taken, so this could be reproduced for the second measurement of the same knee to ensure the same angle was being assessed for intra-observer reliability. After three angles were assessed, each volunteer was asked to maximally actively flex. At this angle a foot bolster was placed on the anterior aspect of the leg in order to maintain this angle for the duration of measurements and prevent change in angle with fatigue (Fig. 2). Finally, the bolster was placed under the leg, just proximal to the ankle and the subject asked to maximally actively extend, and measurements taken (Fig. 3). Active, rather than passive or forced, angles were measured in order to represent clinical application and prevent a difference in force applied by different users.

The recognised method of measuring angles around the knee is to measure the axis of the femur between the centre of the greater trochanter and the lateral epicondyle of the femur, and the axis of the tibia between the lateral femoral epicondyle and the centre of the lateral malleolus (Jones et al., 2014). This method was used for all techniques of goniometry. At each angle, measurements were first estimated visually (VE), then measured using a short arm goniometer (SG), followed by a long arm goniometer(LG). An iPhone 7 Plus (Apple Inc., Cupertino, California) running Goniometer Pro application (5FUF5 CO) on iOS 10.2.1 was used for the smartphone reading (SP). This device uses the internal inclinometer in the smartphone, registers an angle at one screen press and on movement of the phone orientation (axis of this is adjustable) measures the difference between the two angles. The final measurement was taken with the Halo Digital Goniometer (Halo Medical Devices, Sydney, Australia). Each device measures to the nearest whole degree.

**Fig. 4:**
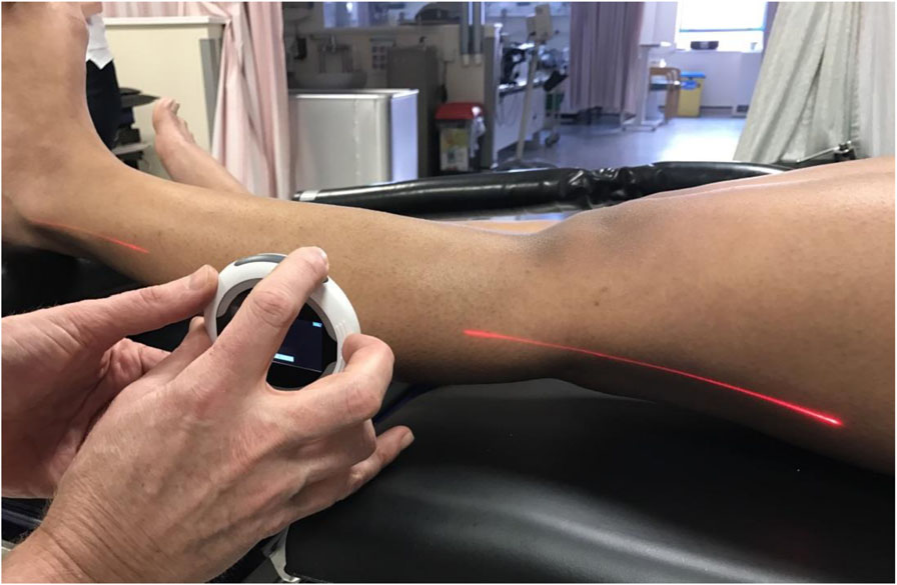
Use of laser projection with Halo Digital Goniometer

**Fig. 5:**
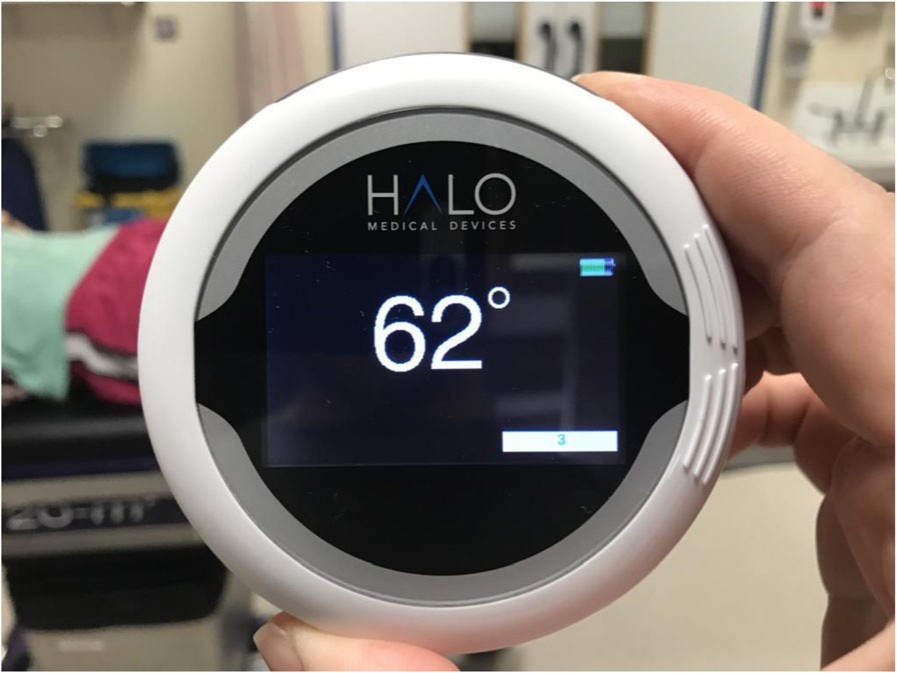
Reading provided after measurement by Halo Digital Goniometer

Despite one study suggesting the optimal position for a smartphone for goniometry measurements around the knee (though using a different application) was on the anterior surface of the thigh, followed by the anterior surface of the leg (Pereira et al., 2017), in our normal cohort of patients in which the majority have a BMI above normal, we do not think this would be most appropriate as this would lead to large variability in position, and also leads to a potential error in differing the measuring technique from the 4 other methods. We therefore placed the long edge of the iPhone along the axis between greater trochanter to lateral femoral epicondyle to lateral malleolus as described above. The Halo Digital Goniometer (HDG) produces a laser beam either side of the device, which was lined up upon the same axes (Fig. 4). Both the smartphone app and the Halo device require a button press to set zero and a second button press when the device has been moved to the second position, the display then produces the angle measured (Fig. 5).

All techniques were performed for each angle by 3 users: one consultant orthopaedic surgeon with a special interest in arthroplasty (KRW), one specialty registrar (resident) in trauma and orthopaedics (GEH) and the lead orthopaedic physiotherapist in our unit (TH). After all methods were used to assess a set angle, the angle was changed as described above, and after all five angles, the subject was changed. One knee was measured in each subject, followed by the contralateral knee in the same order. Each user therefore took 150 readings on the first assessment of each knee. Following this, the users were blinded to their original readings and the process was repeated to perform the second measurement for intra-rather reliability, reproducing the original angles as described. This reduces recall bias due to the significant amount of data points required for each user to recall for this to be a factor, and the time gap between these measurements of approximately 2 h.

Readings were recorded by the users themselves. Visual estimation was always carried out first so as not to be influenced by directly measured angles. For the 2 analogue-type goniometers users were asked to set the angle using the arms of the devices without looking at the reading, to then remove the device and record the result to reduce unblinding bias as much as possible. For the two digital devices, the result is not shown until the final button press and we therefore feel unblinding is not a significant factor. Users were not aware of their corresponding users’ measurements.

This technique led to each user collecting 300 data points. As a result, for inter-rater reliability for each device there were 3 sets of 60 measurements for analysis (5 angles in 6 knees, performed twice producing 60 measurements) and for intra-rater reliability there were 2 sets of 90 measurements (comparison of two measurements of: 5 angles in 6 knees, taken by 3 users, producing 90 measurements).

### Statistical analysis

Intra-class correlation coefficient (ICC) was calculated for both inter-rater and intra-rater reliability from a two-way random effects model. A satisfactory ICC is generally accepted as > 0.70, and excellent > 0.90. Previous studies have used standard error of mean (SEM) to suggest how accurate each method is, however, a Bland Altman plot using 1.96 multiplied by standard deviation (SD) to infer a 95% confidence interval, gives a range in which the true measurement lies, and has been described in previous studies (Ockendon & Gilbert, 2012). We therefore used this to generate a 95% confidence interval for each method, in order to assess accuracy with the minimum significant difference. A one-way ANOVA was performed to compare measurements between the users.

All data was analysed using SPSS software 23.0 (IBM Corporation).

## Results

Analysis for inter-rater reliability demonstrated all 5 techniques had an ICC > 0.99 (VE = 0.991, SG = 0.991, SP = 0.994, LG = 0.996, HDG = 0.999). The Halo device produced a near perfect correlation with 95% CI of 0.999–1.000.

Intra-rater reliability analysis demonstrated similar results, with all techniques > 0.98 (VE = 0.989, SG = 0.986, SP = 0.991, LG = 0.993, HDG = 0.994). This would suggest that all methods have excellent inter-rater and intra-rater reliability.

The results of significance from one way ANOVA are demonstrated in Table 1. No significant difference between users is demonstrated within methods. Using 1.96xSD, each of the techniques had the following minimum significant differences:

- Halo Digital Goniometer 6°
- Long arm goniometer 10°
- Goniometer pro app 12°
- Visual estimation 14°
- Short arm goniometer 14°

**Table 1:** Comparison of minimum significant difference between methods of measurement (standard deviation in degrees between measurements, 1.96 x standard deviation used for minimum significant difference)

User comparison	Standard deviation	*p*	1.96 x SD
Visual Estimation	8.94	0.996	14.31
Short arm goniometer	8.89	0.999	14.23
Long arm goniometer	4.91	0.973	9.62
Smartphone app	5.98	0.966	11.72
Halo Digital Goniometer	2.97	0.983	5.83

(VE placed above SG due to the higher ICC for intra-rater reliability)

## Discussion

Results suggest that the Halo Digital Goniometer is the most useable device for clinical and research purposes as a difference of more than 6° between measurements can be considered significant. All devices demonstrated high inter and intra rater reliability, suggesting that if a single device is chosen for use, measurements between users and from the same user can be compared, provided the minimum significant difference demonstrated is considered.

ICC calculations for both inter-rater and intra-rater reliabilities were very high for all techniques of measurement. This not a clinically applicable analysis and we believe that, due to the large range of measurements, the statistical analysis is affected to give high results for all, regardless of the lack of agreement between results, and this has also been described in a previous study on knee angle measurement (Miner et al., 2003). For example, in VE there was a range of measurements of 15–20 degrees in 1 in 6 angles taken, yet ICC was 0.991 for inter-rater reliability.

Peters et al. (Peters et al., 2011) studied visual estimation, hand goniometry (short arm goniometer in our study) and radiographic goniometry. They assessed significance of differences in measurement in full extension and full flexion between methods, rather than accuracy of each method. They also assessed ICC for each method, finding all to be ≥0.80 except inter-rater reliability for hand goniometry assessment of extension. They also found that comparing across methods gave low ICC values (extension 0.45, flexion 0.52), suggesting, as expected, that different methods of assessment should not be inter-changed.

It was expected in our results that VE would be the least accurate technique of measurement and this proved to be the case. If a single surgeon sees a patient at every appointment they may be able to appreciate without measurement whether range is improving or decreasing, but visually estimating angles does not seem effective for documentation or when other members of staff are involved in care.

The standard, short goniometer, was found to be as inaccurate as visual estimation, and should likely, therefore, be abandoned for measurement of knee angles.

Lenssen et al. (Miner et al., 2003) assessed the use of a long arm goniometer after TKA in hospital, describing different limits of agreement for measurement of flexion versus extension (8.2° vs 17.6° respectively). This is a large variance in minimum significant difference, and when performed for our data the variance is much lower (7.5° vs 10.1°). This paper is different to ours with respect to the subjects used, as we used normal individuals and you would therefore expect a much larger degree of flexion, but the error generated is most similar for flexion. It is probably more useful when performing research/assessing clinical progression to have a single error for the device in use, and as we assessed a large range of differing angles, feel our results are valid. Passive flexion was also used and therefore the force applied by the examiner may differ, whereas our data for full flexion and extension was active and subject controlled, and should therefore be more reliable. The use of orthopaedic patients post-operatively when considering accuracy may also lead to error as they are at risk of fatigue in holding a position of flexion.

The advent of smartphones has led to a number of publications on their use as goniometers; their benefit being that most users will have easy access to a device. Some publications only comment on ICC (Lenssen et al., 2007). Ockendon (Cleffken et al., 2007), however, reported comparison of a smartphone goniometer to the Lafayette goniometer (which is comparable to our long arm goniometer), using a 95% confidence interval and reporting accuracy of the smartphone app to be 4.6° and the Lafayette goniometer to 9.6°. The results here for the smartphone app demonstrate greater accuracy than our results. They did, however, only measure angles between 5 and 45 degrees, which may affect the overall accuracy.

A recent study (Pereira et al., 2017) comparing visual estimation, a long arm goniometer and a smartphone app demonstrated no significant difference between the experience of the user, and commented that there was a high level of consistency for all methods with ICC = 0.94. High reliability correlation has also been reported using smartphone applications utilising repeated photographs with subsequent measurement of angle (Ferriero et al., 2013), but this process is not quick for use in every patient seen in a clinic.

A significant limitation of smartphone apps is the rapid development and change in both hardware and software. Both of the above studies used differing models of Apple iPhone (3GS and 5), and our study used iPhone 7 Plus. Software and hardware change leads to inherent error. The use of any other brand of hardware or software may also lead to further error. In our experience of their use in our study, the smartphone was subjectively relatively difficult to align to the correct axis, and required direct placement onto the subject’s leg. If using a smartphone in clinical practice, there is a potential infection risk involved with this, unless appropriately covered. The purchase of a smartphone solely for use as a goniometer is also relatively expensive, and this would be required to attempt to negate the error of differing hardware and software. Given the superiority in reliability, availability, and cost of a long arm goniometer in comparison to the smartphone app tested, we could not advocate the use of a smartphone app.

The Halo Digital Goniometer was found to have the smallest minimum significant difference of 6°. This would suggest that for the purposes of research and monitoring that it is the most reliable tool for knee angle measurement. The learning curve for use was very short, and due to the laser projection, did not require any direct contact with the patient, which is an advantage for infection risk, especially if there is a desire to measure range of motion intra-operatively. Measurement could also be performed with a single hand, leaving the other hand of the user free to support the patient, or palpate the appropriate landmark if required. It must be clarified that the manufacturer of this device states an accuracy of their device to 1° for angle measurements, we demonstrate that for knee measurements, there is a 6° difference required between two measured angles to be sure of a significantly different angle.

We feel that this is one of the most in-depth and clinically applicable studies into knee goniometry. There are few clinical studies of knee goniometry that have used surgeon, surgical trainee and physiotherapist for measurements, and it has previously been commented that the ideal would be to include all staff types (Pereira et al., 2017; Miner et al., 2003). By using staff from all grades who assess patients in clinic and post-operatively, our data is more robust. Our data is also of greater volume than previous studies (Ferriero et al., 2013; Jones et al., 2014; Cleffken et al., 2007; Lenssen et al., 2007; Peters et al., 2011).

A potential limitation of our study is that we did not compare between similar experience levels, for example, between two physiotherapists. This was not done for two reasons. Firstly, previous published data suggests no significant difference in measurements taken by similar staff groups (Pereira et al., 2017). Secondly, prior to collection of this data, we performed a pilot study, using two specialty registrars, two physiotherapists and two medical students, where there was no significant difference between measurements taken in any group. It was therefore felt that a reduced number of users, spanning all staff types and collecting a greater number of data points would be more beneficial. A further limitation is the lack of comparison to a ‘gold-standard’ or use of radiographic analysis and therefore an absolute accuracy of each device cannot be given, although inaccuracies are also present in the use of radiographs, and exposure of subjects to radiation for the purpose of assessing goniometry devices was felt to be excessive. It may also be beneficial to have a larger time gap between measurements, or to perform the same data collection on a different day, but this was not logistically possible.

## Conclusion

This study demonstrates that the Halo Digital Goniometer is the most reliable of all devices assessed with the smallest minimum significant difference of measurements. Where two angles are taken, this device can show a definite difference between measurements, providing the measured difference is greater than 6°. This device has an added advantage of being non-touch to the patient, and single hand use.
